# Cross-Platform Ecological Momentary Assessment App (JTrack-EMA+): Development and Usability Study

**DOI:** 10.2196/51689

**Published:** 2025-01-28

**Authors:** Mehran Sahandi Far, Jona M Fischer, Svea Senge, Robin Rathmakers, Thomas Meissner, Dominik Schneble, Mamaka Narava, Simon B Eickhoff, Juergen Dukart

**Affiliations:** 1 Research Centre Jülich Institute of Neuroscience and Medicine Brain and Behaviour (INM-7) Jülich Germany; 2 Medical Faculty Institute of Systems Neuroscience Heinrich Heine University Düsseldorf Düsseldorf Germany; 3 Department of General Paediatrics, Neonatology and Paediatric Cardiology Medical Faculty University Hospital Düsseldorf, Heinrich-Heine-University Düsseldorf Germany

**Keywords:** digital biomarkers, mobile health, remote monitoring, smartphone, mobile phone, monitoring, biomarker, ecological momentary assessment, application, costly, user experience, data management, mobility

## Abstract

**Background:**

Traditional in-clinic methods of collecting self-reported information are costly, time-consuming, subjective, and often limited in the quality and quantity of observation. However, smartphone-based ecological momentary assessments (EMAs) provide complementary information to in-clinic visits by collecting real-time, frequent, and longitudinal data that are ecologically valid. While these methods are promising, they are often prone to various technical obstacles. However, despite the potential of smartphone-based EMAs, they face technical obstacles that impact adaptability, availability, and interoperability across devices and operating systems. Deficiencies in these areas can contribute to selection bias by excluding participants with unsupported devices or limited digital literacy, increase development and maintenance costs, and extend deployment timelines. Moreover, these limitations not only impede the configurability of existing solutions but also hinder their adoption for addressing diverse clinical challenges.

**Objective:**

The primary aim of this research was to develop a cross-platform EMA app that ensures a uniform user experience and core features across various operating systems. Emphasis was placed on maximizing the integration and adaptability to various study designs, all while maintaining strict adherence to security and privacy protocols. JTrack-EMA+ was designed and implemented per the FAIR (findable, accessible, interpretable, and reusable) principles in both its architecture and data management layers, thereby reducing the burden of integration for clinicians and researchers.

**Methods:**

JTrack-EMA+ was built using the Flutter framework, enabling it to run seamlessly across different platforms. This platform comprises two main components. JDash (Research Centre Jülich, Institute of Neuroscience and Medicine, Brain and Behaviour [INM-7]) is an online management tool created using Python (Python Software Foundation) with the Django (Django Software Foundation) framework. This online dashboard offers comprehensive study management tools, including assessment design, user administration, data quality control, and a reminder casting center. The JTrack-EMA+ app supports a wide range of question types, allowing flexibility in assessment design. It also has configurable assessment logic and the ability to include supplementary materials for a richer user experience. It strongly commits to security and privacy and complies with the General Data Protection Regulations to safeguard user data and ensure confidentiality.

**Results:**

We investigated our platform in a pilot study with 480 days of follow-up to assess participants’ compliance. The 6-month average compliance was 49.3%, significantly declining (*P*=.004) from 66.7% in the first month to 42% in the sixth month.

**Conclusions:**

The JTrack-EMA+ platform prioritizes platform-independent architecture, providing an easy entry point for clinical researchers to deploy EMA in their respective clinical studies. Remote and home-based assessments of EMA using this platform can provide valuable insights into patients’ daily lives, particularly in a population with limited mobility or inconsistent access to health care services.

## Introduction

Self-reported information in psychiatric disorders plays a crucial role in clinical decision-making. They are often gathered through traditional questionnaires or verbal conversations during clinic visits [1]. These visits are infrequent, costly, and inaccessible to everyone [2]. The self-reports from in-clinic visits may also be subject to higher recall bias, notably when asked to recall memories and past events [3].

The need to reduce reliance on traditional in-clinic visits and the widespread adoption of smart devices (such as smartphones and smartwatches) have increased their potential for use in clinical settings. Health-related data collected from these devices are often called digital biomarkers (DBs) [4-6]. DBs include a wide range of measurements, with ecological momentary assessments (EMAs) being one of the most widely accepted and used [7]. EMA involves using experience sampling methods, which are brief assessments administered in real time in naturalistic settings to study the moment-to-moment fluctuations of states and behaviors [8]. EMA offers several advantages over traditional in-clinic visits, including a higher sampling frequency, the ability to collect longitudinal data, enhanced engagement, lower costs, and increased ecological validity [1,9]. Smartphones are often equipped with a range of sensors such as location, pedometer, proximity, and luminance sensors. These sensors can be used to trigger surveys based on different physical activities or environmental factors. For instance, a survey can be automatically triggered when the user visits new locations or there is a change in weather conditions in the user’s location. This type of EMA is commonly referred to as event-contingent EMA [10,11].

Assessment questions, supporting material (eg, images, videos, and explanatory text), managing assessment logic (ie, scheduling, inclusion or exclusion criteria, validating input answers, recording timestamps, and storing and transferring assessments), and providing assessment completion reminders [12] are among the main features typically integrated into EMA apps. These advanced capabilities offer an excellent opportunity for collecting DBs in remote assessments. However, their implementation comes with several technical challenges and obstacles. These challenges include but are not limited to privacy and security, memory and battery usage, patient acceptance, scalability, and interoperability with different devices and operating systems. Although there are various apps intended to meet many of the abovementioned criteria [13-17], interoperability with different operating systems, adaptability, and configurability are still lacking. Several operating systems are available for mobile (Android and iOS) and desktop (Windows and macOS) devices. Each of these has its own paradigms and programming languages. Therefore, separate developers and experts are required for each, significantly increasing the cost and development time. Additionally, restricting the apps to a specific operating system may lead to a selection bias. A platform-independent architecture addresses these limitations. This motivated us to introduce JTrack-EMA+ as a cross-platform, configurable app that also complies with the General Data Protection Regulation (GDPR). It consists of an EMA app and an online management tool facilitating assessment management and data quality control. It is integrated into our previously introduced sensor-based DBs ecosystem, “JTrack” [18]. In this paper, we also evaluated a pilot study using JTrack-EMA+ in a population of parents with a newborn to investigate participants’ compliance with the assessment protocols.

## Methods

### Study Design

**Figure 1:**
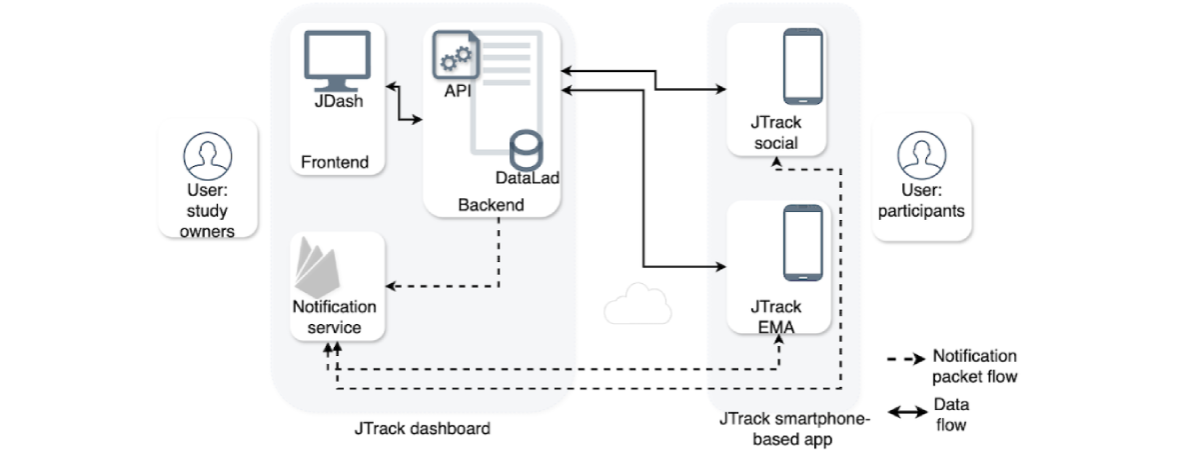
JTrack platform’s main structure and its working principle. The flow of data packages between JDash and the JTrack app and the flow of push notifications between servers and the app are shown here. API: application programming interface; EMA: ecological momentary assessment.

**Figure 2:**
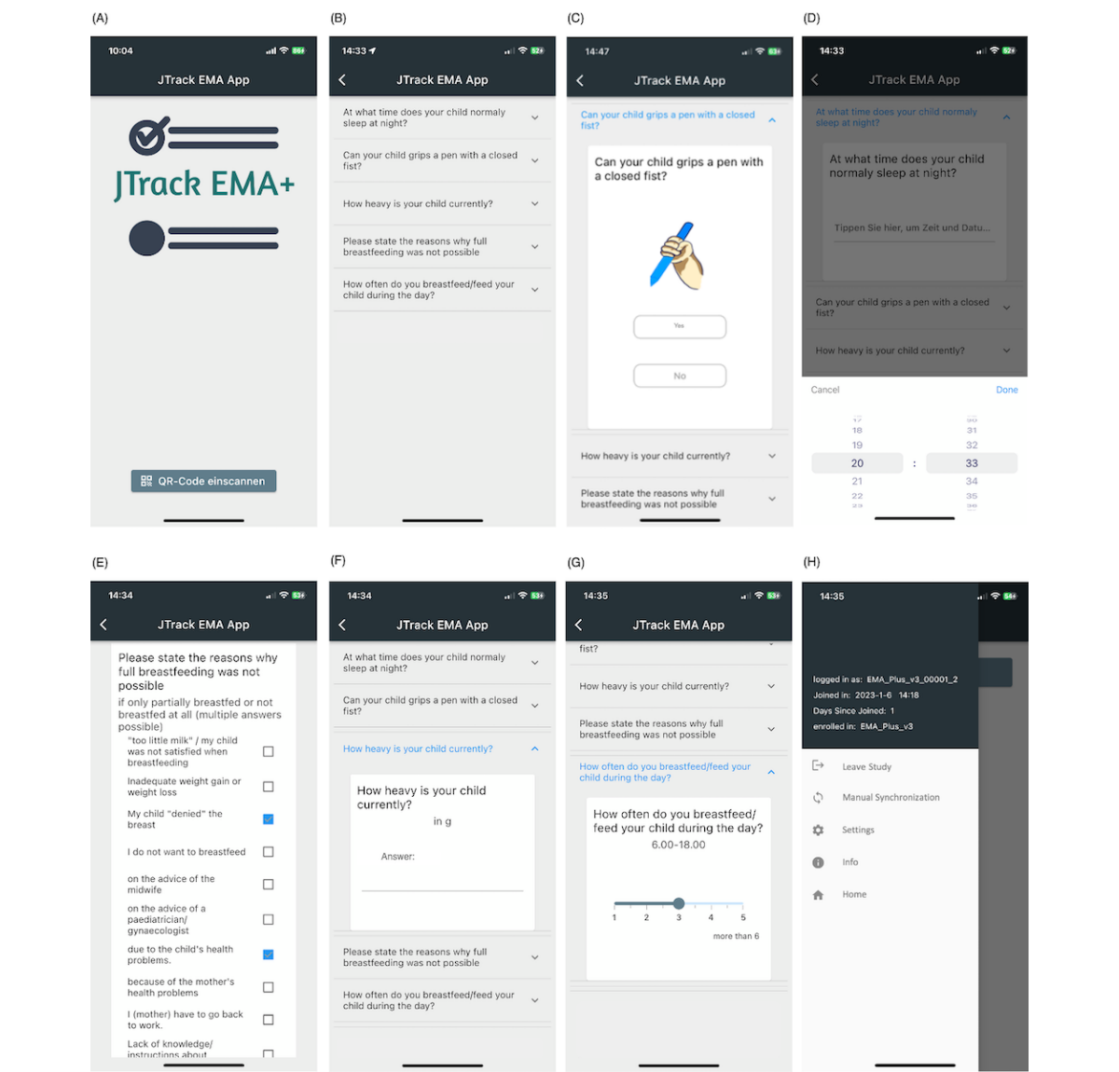
JTrack-EMA+ smartphone app user interface. (A) Initial screen to scan a QR code used to join a study; (B) list view page to see all available questions; (C) binary question with supporting image; (D) time and date question; (E) multiple selection question; (F) user numeric input question; (G) sliding question; and (H) main menu to access leaving, manual synchronization, and setting pages. EMA: ecological momentary assessment.

**Figure 3:**
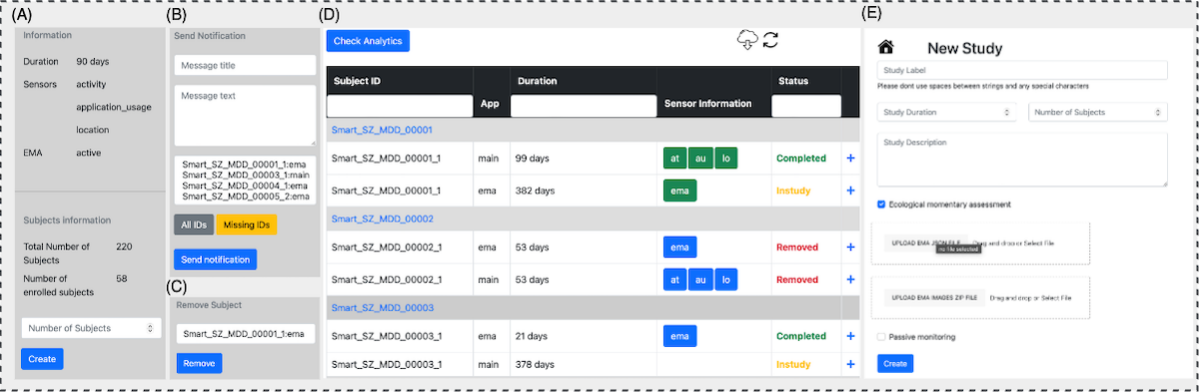
JDash user interface. (A) Display general information about the current study; (B) manage and send push notifications to participants; (C) remove a participant from the study; (D) display the main screen, which visualizes the supporting information for enrolled subjects in the study—it also color codes the status of participants (ie, left the study, have missing data, etc); and (E) create a new study, and add questions list or images and details of the study. EMA: ecological momentary assessment.

**Figure 4:**
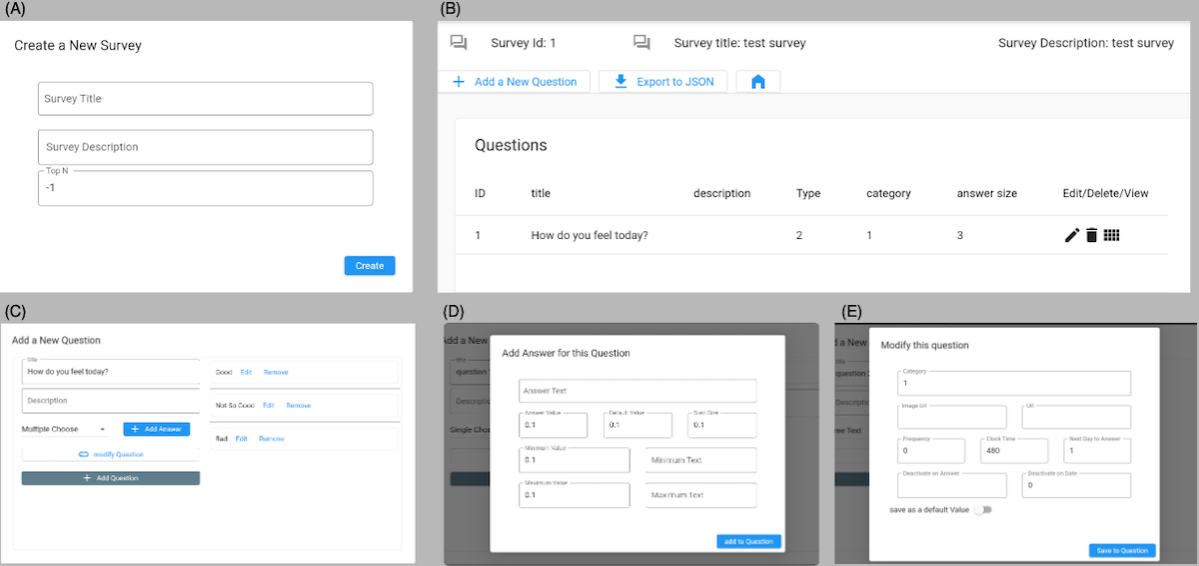
JDash EMA creator user interface, integrated into JDash. (A) Create a new assessment, (B) display the main screen of the assessment and related information, (C) add a new question to the assessment, (D) add answers to the question from the previous step, and (E) modify and add logic to the question. EMA: ecological momentary assessment.

Here, we describe the primary component of the JTrack platform (Figure 1), which includes a smartphone-based app JTrack-EMA+ (Figure 2A-H), and an online management app, JDash (Figures 3A-E and 4A-E). JTrack-EMA+ was developed based on platform-independent architecture using the Flutter framework [19]. Flutter is a cross-platform framework developed by Google that allows developers to build native apps from a single codebase. There are different cross-platform technologies such as React Native (Meta Platforms, Inc) and Xamarin (Microsoft). However, Flutter has several advantages that make it an irresistible choice for JTrack-EMA+. Flutter uses its rendering engine, which provides better performance and a more consistent look and feel across platforms compared to React Native, which relies on native components. Flutter’s hot reload feature enables faster iteration and testing during development, improving the overall development workflow and Flutter’s widget-based approach to user interface development promotes a consistent and cohesive user experience across platforms. Additionally, Flutter has a more active and growing community, with strong support from Google, ensuring continued development and improvement of the framework.

Platform-independent design reduces development and maintenance time, provides a consistent performance and user experience, and enables instant delivery of new feature updates and enhancements across multiple platforms.

### Adaptability and Configurability

The behavior and performance of software are significantly impacted by rapidly evolving operating systems. Being able to adapt to these changes in agile is a crucial trait that the platform-independent architecture of JTrack-EMA+ provides. Besides architectural benefits, JTrack-EMA+ constructs evaluation materials and logic using a JSON file to facilitate reusability and configurability.

The use of a JSON file providing the configurations for each specific study can simplify the process of reusability in an app by centralizing configuration fields in a single file. This approach allows for efficient management and modification of app settings without the need to alter the codebase. By storing configuration details such as logging credentials, user and study IDs, and survey-related data in a JSON file, study owners can quickly update these settings as needed without modifying the app code. This approach enhances flexibility, scalability, and maintainability by separating configuration from the app logic. It makes it easier to reuse the same configuration across different environments or instances of the app. Using such a configuration file approach, study owners can modify the app settings from the study management interface without further need to alter the app codebase, making it easier to manage and maintain.

After users register with the provided QR codes, the assessment is generated based on a study-specific JSON file received from the server. Additionally, assessments can be updated remotely using this file without updating the app. The browser-based study management dashboard JDash has a built-in EMA creator to generate these JSON files making the design and configuration of an EMA study quite simple and versatile (Figure 4).

JTrack-EMA+ supports a wide range of assessment formats, such as binary, multiple-choice, text, or numeric user input; date and time; and sliding questions (Figure 2). The system has a flexible assessment logic that includes conditions, categories, and customizable frequency on a question-by-question basis. This functionality is vital in determining whether to ask or skip certain questions, based on predetermined criteria such as the time elapsed since the assessment began, the present time of day, and the value or date of the participant’s response (conditional questions). By categorizing questions, different conditions can also be applied within each category (ie, only show the first n questions for each category). Additionally, the system allows for defining the frequency at which the questions should be displayed to users (such as on-demand, daily, or weekly).

JTrack-EMA+ supports two distinct paging styles: page view and list view. With page view, participants will see a single question at a time, whereas list view shows all questions as a list, allowing participants to expand (choose) a question to answer. While page view ensures the visibility of each question, list view is especially helpful in situations where many questions are shown or only specific questions are relevant.

### Data Quality, Privacy, and Security

Quality, security, and privacy of the collected data are important considerations when designing a novel EMA app. To address the data quality, syntactic input validation is deployed to ensure that only properly formed data is entered. Additionally, to prevent possible data loss, we adopted the “local-first” data storage method, where data are stored locally on the device and only synced to the server when connected to the internet. In addition, to mitigate the impact of potential interruptions (eg, by other programs, battery drain, or phone restart) on saving data, we implemented an auto-saving mechanism.

To ensure data privacy and security and to comply with the European Union GDPR, JTrack-EMA+ deployment does not require any identifiers or personal data. All data are assigned to a JDash-generated user ID allowing for fully anonymized and pseudonymized data collection. Secure channels are used for communication between servers and devices, and data integrity is protected against unintended corruption and potential interruptions using message-digest algorithms. Moreover, the distribution of the app through official channels (ie, Google Play Store and Apple App Store) provides convenience to the participants and creates a sense of security and confidentiality.

QR code-based registration, embedding control, and authentication methods streamline the registration process (these codes are generated when a study is created in JDash). In addition, we have provided a “leave study” option in the app, which enables all participants to withdraw from the study at any time.

### Study Management

JDash is an online study management app designed to assist JTrack study owners in creating new studies and managing ongoing ones (Figure 3A-E). It allows study owners to create and modify all study aspects, such as the duration, addition of assessment materials (eg, images), data quality checks, removal of participants from a study, ending of a study, and management of the study owner’s access level. Python (Django and Web Server Gateway Interface) was chosen as the implementation technology. JDash consists of a user interface and a backend. While the backend app programming interface serves as an end point for receiving, saving, and authentication requests arriving from the app, the frontend interface can also be used to create and design an assessment (Figure 1).

The JDash was developed to be easy to use and deploy. It incorporates flawless design principles to provide a range of features for clinicians to establish their assessments intuitively. The design principles used in the app ensure that clinicians can navigate and use the assessment tools efficiently, without further help from a technical team, enhancing the overall user experience. By focusing on such design principles, the app aims to streamline the assessment process for clinicians, making it user-friendly, clear, and consistent (eg, similar design patterns). This approach aligns with best user interface design and usability practices, ultimately benefiting users by simplifying their workflow and improving the effectiveness of their assessments.

In addition to an open-source software license, JDash uses an automated deployment model that is efficient, easy to use, and requires fewer configurations (for instruction on deployment, readers may refer to the documentation on our GitHub page [20]). In addition, JDash has a comprehensive notification system to ensure periodic reminders based on a predetermined schedule, avoiding unwanted (eg, overnight) alerts. This notification is designed to operate as a push notification (online) that can be sent to participants’ devices from the dashboard. This module is integrated into JDash to deliver reminders to participants’ devices, either individually or based on their status (ie, no data sent in a few days). This regular reminder has the potential to minimize the high dropout rate of EMA-based assessments [9].

Finally, most research programs aim to share data with colleagues or along with research results to promote transparency, encourage collaboration, and accelerate research. To facilitate this goal, we have used DataLad [21] as a data management infrastructure on our servers to manage data according to FAIR (findable, accessible, interpretable, and reusable) principles [22]. DataLad offers essential features such as data versioning, metadata handling, structured formatting, and change tracking under an open-source license which contributes to enhanced security and privacy of collected data. This integration in the data management layer also accelerates reproducible science, a key element of scientific methods.

### Pilot Study

We deployed the JTrack-EMA+ app in a pilot study involving 179 parents with newborns followed up for 480 days. Parents were asked to install the JTrack app on their phones (75 iOS and 104 Android). The pilot study includes weekly assessments of children’s physical and mental development. These questions are designed to appear based on their defined frequency and the time since participants joined the study. Furthermore, the yes or no questions deactivate when the answer is “Yes.” To illustrate the development questions, a picture was shown for each question. Martin Pawlusiak designed the pictures. Additionally, an appropriate notification time was chosen to avoid receiving assessment reminders early in the morning or late at night.

We have been conducting the pilot study since November 2021, and it is set to run until November 2023. As of now, we have released the preliminary compliance data that we have gathered over the first 14 months of the study. We calculated the compliance rate as a percentage of completed surveys compared to what was expected. In total, 65 participants completed assessments for at least 6 months and were included in the compliance analysis. Monthly compliance was computed as the number of received data points divided by the number of expected data points. Monthly average compliance was compared using the Friedman test, followed by pairwise comparisons using the Wilcoxon rank sum test.

### Ethical Considerations

The studies involving human participants were reviewed and approved by the ethics committee at the medical faculty, Heinrich-Heine-Universität Düsseldorf, Germany (2021-1500_1). The patients or participants provided written informed consent to participate in this study. The data were anonymized. Participants did not receive any compensation for this study.

## Results

**Figure 5:**
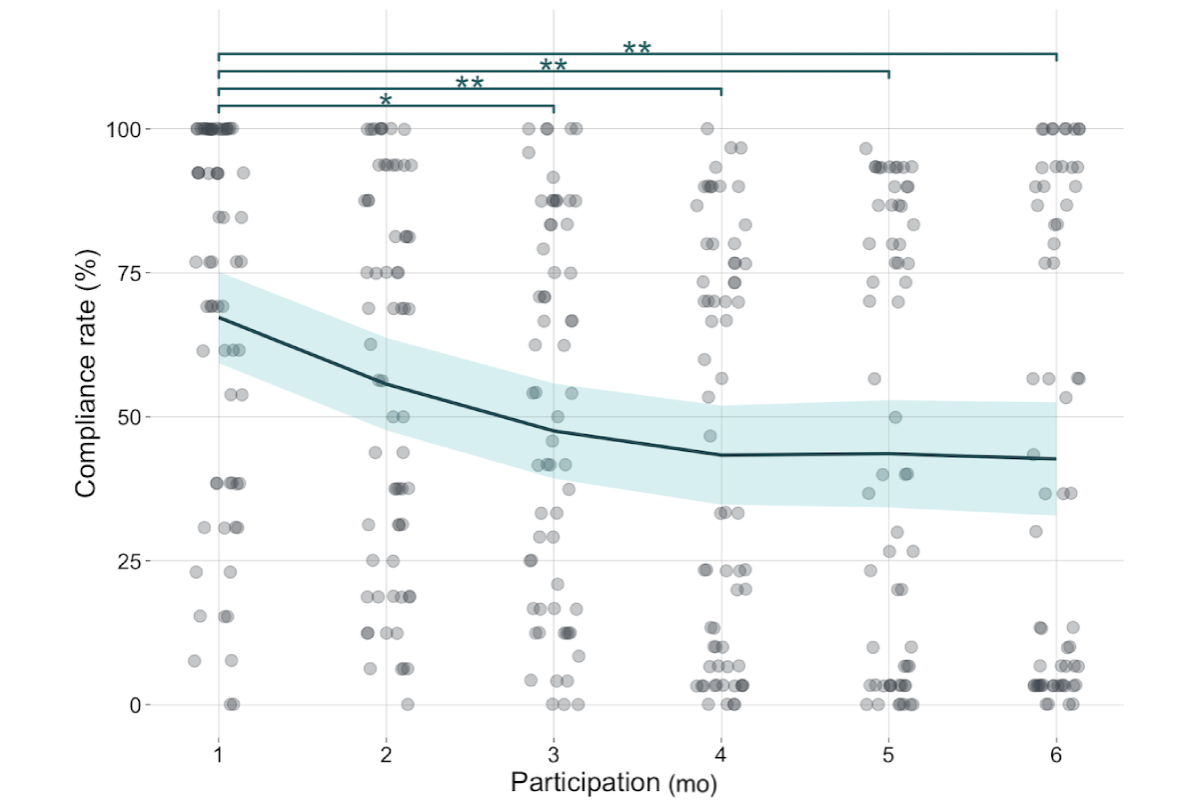
Compliance rate for an exemplary study. Each dot represents a participant with a corresponding compliance rate (calculated as a percentage of completed surveys out of expected surveys), and a solid line is the average compliance for a corresponding month with a 95% CI. *P<.05, **P<.01.

Figure 5 displays the fluctuation of average compliance rates across the 6 months of the pilot study. The overall average compliance rate across this period was 49.3%. The between-group comparisons of the average compliance rate show a significant decrease from 66.7% in the first month to 46.9% (*P*=.01), 42.6% (*P*<.001), 42.9% (*P*=.002), and 42% (*P*=.004) in the third, fourth, fifth, and sixth months, respectively.

## Discussion

### Principal Findings

Here, we introduced JTrack-EMA+, a smartphone-based, real-time EMA app. This platform-independent architecture of the app reduces the disparity in user experiences and functionality across different operating systems. It complies with the GDPRs and official app distribution channels while providing all features required for designing and deploying EMA-based evaluations.

The online dashboard serves as a central study management interface. It visualizes and summarizes the study information (ie, duration, the number of subjects, and supplementary information). This information is used for data streaming and quality checks and to interact with participants in case of missing or invalid data via push notifications. Researchers can control the access to the specific information of the study to their research partners through the user access level control provided by the dashboard. The open-source solution DataLad [21] is used for data management in JDash, and the abovementioned functionalities reduce reliance on third-party apps and services such as Amazon Web Services.

The traditional development architecture used by existing platforms is limited by its shortcomings [13-17]. A lack of support and disparities in user experience and app functionality across platforms are among the most significant contributors. Therefore, most EMA platforms are either available for a single operating system (ie, ULTEMAT is only available for Android [16], and iHabit is only available for iOS [17]) or have different features depending on the operating system (ie, AWARE, Remote Assessment of Disease and Relapse–base, and SEMA3 have two distinct apps for each platform [13,15]).

To the best of our knowledge, the JTrack-EMA+ stands out as a pioneer in prioritizing platform-independent architecture. This platform offers extensive configurability, which sets it apart from the existing platform. It also provides a flexible and dynamic environment instead of being limited to a single problem-oriented solution.

The effectiveness of EMA depends on the participants’ motivation and adherence to assessment protocols [12,23]. Poor compliance and missing data can adversely impact the quality of the collected data and introduce bias in the sample, thereby hindering the success of clinical apps. Our results revealed an overall average of 49.3% with a significant decrease in compliance rate after 2 months. Although there is a large degree of heterogeneity in compliance rates reported in various studies [24], considering our inclusion time constraint (6 months) and the target demographic (parents with busier routines who dedicate more time to their newborn), this compliance rate can be considered an acceptable rate. Importantly, there is no definitive recommendation for a minimum or appropriate rate of compliance as it is highly dependent on the specific patient or participant population and the actual frequency, duration, and comprehensiveness of the specific assessments, as well as the actual research question. As long as sufficient representation of the individual day-to-day variability in the respective EMA assessments is achieved, the respective EMA measures could be considered useful. Overall, our results demonstrate the potential to use our platform compared to the paper-and-pencil EMA assessment (which was shown to have a significantly lower compliance rate), particularly in participants with limited mobility or restricted access to health care services [24].

A limitation of our platform is its ability to detect and respond to various events and contexts, such as environmental and physical activities. Although we consider this a limitation here, it is carried out on purpose to simplify the ethics and data privacy required by sensory and contextual assessment; required in addition to EMA. Additionally, despite the attempts to keep performance and user experience consistent across all operating systems, background work constraints in iOS may influence the app’s internal reminders on these devices, so automated push notifications implemented by JDash can be a better choice for such devices.

Finally, JTrack-EMA+ is an active project regularly updated to add new features and improve the platform’s functionality. The features described here are part of the version 1 release. Therefore, later versions may have additional functions which will be documented with the respective releases. The most updated JTrack-EMA+ versions are released via the official distribution channels (ie, Google’s Play Store and Apple’s App Store). All study management functionalities to deploy JTrack-EMA+ are implemented as open source and can be accessed from our GitHub repository under an open-source license.

### Conclusions

In this paper, we covered the key concepts associated with EMA apps and the technical constraints of existing platforms. Then we launched a platform-independent JTrack-EMA+ app to reduce the gap in user experience and app functionality across different operating systems while adhering to security, privacy, and GDPR requirements.

In a pilot study, we also demonstrated participants’ compliance rate with assessment protocols and how it fluctuated over a 6-month course of study. We conclude that remote and at-home assessment using this platform results in an acceptable rate of compliance that may not be possible using the conventional in-clinic visit method, particularly in a population with limited mobility or a busy schedule—as were the participants in our pilot study.

Incorporating EMA apps into clinical practice can significantly improve patient monitoring in real time, reaching a wider population and enhancing ecological validity. It can lead to personalized treatment interventions by providing comprehensive and dynamic insights into individuals’ health behaviors and experiences. JTrack-EMA+ was originally designed for clinical studies, but it can also be used in different areas where EMA measures may be required such as market research, education, government, public policy, event planning, and feedback and engagement. While using smartphone-based EMA technologies offer many potential benefits, certain populations may face barriers to effectively accessing and using these apps. Some key challenges include access to smartphones or the internet in underresourced regions, older adult individuals, individuals with disabilities, and low digital literacy. A multipronged approach is needed to address these challenges, such as providing alternative low-tech EMA options, designing highly accessible and intuitive interfaces, improving digital literacy through training, and ensuring affordable access to required technologies. In this regard, the future version of JTrack-EMA+ will improve integration for web-based users as an alternative to smartphone-based EMA. Additionally, it will include conditional assessment support and additional features to analyze the collected data directly in a browser-based dashboard (JDash).

Finally, we concluded that platform-independent architectures provide comparable functionality and user experience and save development and maintenance costs over time. These principles are critical to EMA-based assessments’ effectiveness and applicability in clinical trials.

## Abbreviations

DB: digital biomarker
EMA: ecological momentary assessment
FAIR: findable, accessible, interpretable, and reusable
GDPR: General Data Protection Regulation

## Data Availability

On the iOS platform, this app is published as JTrack-EMA.
